# The influence of lime type on the properties of traditional lime-soil materials

**DOI:** 10.1371/journal.pone.0339877

**Published:** 2026-01-16

**Authors:** Shiqiang Fang, Wenjing Hu, Xueqiang Chen, Lina Xie, Luxiang Cai

**Affiliations:** 1 Centre for the Protection of Cultural Property, Ningbo University of Finance & Economics, Ningbo, P.R. China; 2 Department of Cultural Heritage and Museology, Zhejiang University, Hangzhou, P.R. China; 3 School of Art and Design, Ningbo University of Finance & Economics, Ningbo, P.R. China; Jazan University College of Engineering, SAUDI ARABIA

## Abstract

The conservation of historic earthen structures requires repair materials that are both high-performance and historically appropriate. While archaeological evidence indicates that the historical use of blocky quicklime to make lime-soil materials, modern research and conservation practice often focus on its powdered counterpart, creating a knowledge gap. Thus, this work systematically evaluated the physical properties, microstructure, and composition of lime-soil materials prepared with blocky quicklime, powdered quicklime, and hydrated lime (at 10–20% content). The results demonstrated that hydrated lime yielded inferior strength and durability. Although both types of quicklime enhanced performance, they functioned through distinct mechanisms. Powdered quicklime provided consistent, dosage-dependent improvement, making it reliable for high-performance requirements. In contrast, blocky quicklime exhibited a pronounced optimum at 15% content; beyond this, performance declined due to expansion stresses from excess, partially hydrated cores, as identified by FT-IR spectroscopy. This study concluded that the choice of lime form was critical: powdered quicklime was recommended for predictable, broad-spectrum enhancement, whereas blocky quicklime could be effective but required strict dosage control to harness its unique potential and avoid damage. These findings provided a scientific basis for selecting and applying lime materials in the repair of earthen architectural heritage.

## Introduction

Lime-soil materials, which use lime as a binder, have been utilized globally in construction for millennia. In China, numerous architectural relics were built with these materials, including over 400 sites under national-level protection [1]. However, these cultural heritage structures are constantly exposed to natural elements, leading to progressive deterioration and posing significant challenges for their conservatio.

Studies have demonstrated that using traditional lime-soil materials for restoration offers both harmonious visual integration and better physico-chemical compatibility with the original fabric, making them a preferred choice for heritage preservation [2–4]. Thus, these materials have regained research attention, with studies focusing on archaeological analysis of ancient lime-soil samples [5–8], mechanisms of additive action and material degradation [9–12], traditional formulations and performance [8,13,14], and modern optimization approaches [15–17].

**Fig 1:**
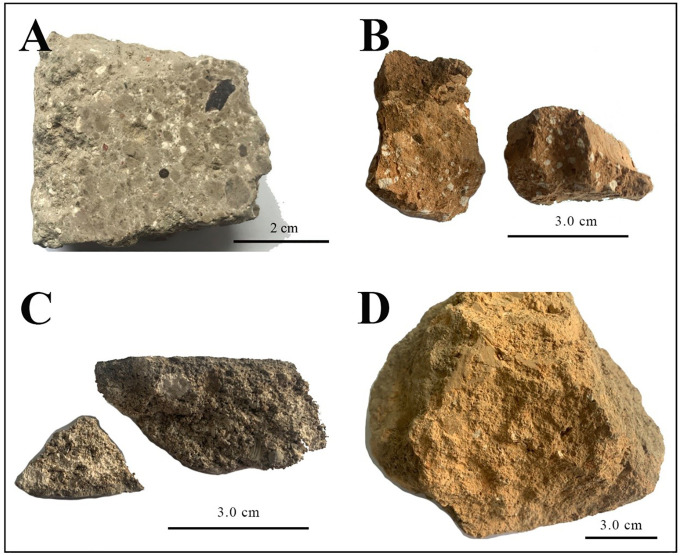
Lime-soil archaeological samples (A. lime-soil used for a tomb, *Li Hongzhang’s* family tomb, Hefei, Anhui, China [30]; B. lime-soil used for masonry, *Lanxi Zhuge Bagua* Village, Jinhua, Zhejiang, China; C. lime-soil used for a floor, *Ningbo Maoxing* Village, Ningbo, Zhejiang, China; D. lime-soil used for a fort, *Ningbo Zhenhaikou* coastal defense, Ningbo, Zhejiang, China).

In the preparation of lime-soil materials, quicklime generally exhibits superior performance to hydrated lime [13,18]. This is mainly attributed to its higher chemical reactivity and the heat released during hydration, which promote ion exchange reactions and facilitate the formation of cementitious products (such as calcium silicate hydrate gels) [19–21]. These processes enhance the material’s mechanical properties and durability. The direct mixing of quicklime with sand, volcanic ash, ceramic fragments and so on, known as the “hot mixing” method [22,23], was common in traditional practice. Archaeological studies of historic lime-based materials often reveal white lumps (Fig 1) [24–26], which are referred to as blocky quicklime in this study. These residual particles suggest that blocky quicklime was used directly in historical construction, which may have been incompletely dissolved or mixed, derived from under- or over-burned limestone, or partially carbonated before use [27–29].

Wichterlová et al. [31] studied the decomposition process of lump quicklime, elucidating its reaction mechanisms and potential effects on carbonation and hardening in lime-based systems. Bakolas et al. [32] suggested that lime lumps in historic mortars may improve mechanical behavior and adaptability within masonry. Similarly, Seymour et al. [22] regarded that such lime lumps could provide a long-term source of reactive calcium, enabling self-healing through pore and crack filling. However, Lee et al. [33] reported that mortars made with powdered quicklime exhibited better mechanical performance than those with traditional lump quicklime, a difference potentially influenced by mixing methods or aggregate properties. To date, however, most related research has focused on historic mortars, with limited attention given to the role of blocky quicklime in lime-soil systems.

On the other hand, although lime-soil materials have been extensively studied in modern engineering, these studies predominantly use finely crushed or powdered quicklime [13,20,21,34–36], with few systematic investigations on blocky quicklime [37]. Moreover, contemporary formulations typically contain lime contents within the 10% to 30% range [34,38–41], while historical lime-soil structures often had much higher proportions, sometimes exceeding 50% [42]. As a result, current studies may not accurately represent traditional lime-soil materials. Therefore, further research on lime-soil composites prepared with blocky quicklime is essential to support the conservation of architectural heritage.

Therefore, this study aims to evaluate and compare lime-soil materials prepared using blocky quicklime, powdered quicklime, and hydrated lime. By analyzing temperature evolution, cohesion, maximum dry density, mechanical properties, and durability, we seek to clarify the influence of lime type on lime-soil material performance.

## Experimental design

### Materials

**Fig 2:**
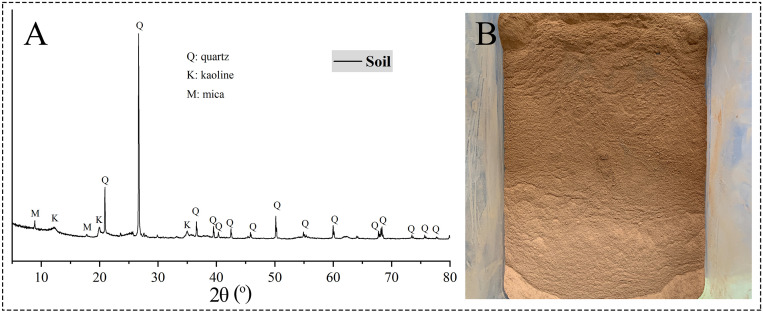
XRD pattern (A) and photograph (B) of the soil used in the study.

The soil used in this study was collected from Ningbo, Zhejiang, China. It was air-dried for 20 d, crushed, and sieved to remove gravel and plant residues. The fraction with a particle size of less than 0.25 mm was retained for sample preparation (Fig 2B). X-ray diffraction (XRD) analysis revealed that quartz was the dominant phase, followed by kaolinite and mica (Table 1, Fig 2A). The elemental composition, as determined by X-ray fluorescence (XRF), was dominated by silicon, aluminum, and iron (Table 1).

**Table 1:** Chemical and mineralogical composition of the raw materials used in this study.

Category	Parameter	Blocky quicklime	Hydrated lime	Powdered quicklime	Soil
**Elemental Composition** **(XRF %)**	**Ca**	77.81%	64.71%	75.72%	–
	**Si**	–	–	–	29.66%
	**Al**	–	–	–	11.98%
	**Fe**	–	0.04%	0.05%	4.97%
	**Ti**	–	–	–	0.69%
	**LE (mainly Oxygen)**	22.02%	35.20%	24.16%	52.39%
	**Total**	99.83%	99.95%	99.93%	99.69%
**Phase Composition** **(XRD wt%)**	**Calcium Oxide**	86.3% ^a^	0%	84.8% ^a^	–
	**Calcium Hydroxide**	13.7% ^a^	100%	15.2% ^a^	–
	**Quartz**	–	–	–	82.7%
	**Kaolinite**	–	–	–	9.4%
	**Mica**	–	–	–	7.9%

Remark: a. The content was calculated as follows: CaO (%) = I _CaO_/(I _CaO_ + I _Ca(OH)₂_) × 100%; Ca(OH)_2_ (%) = I _Ca(OH)₂_/(I _CaO_ + I _Ca(OH)₂_) × 100%, where I is the intensity of the main peak.

**Fig 3:**
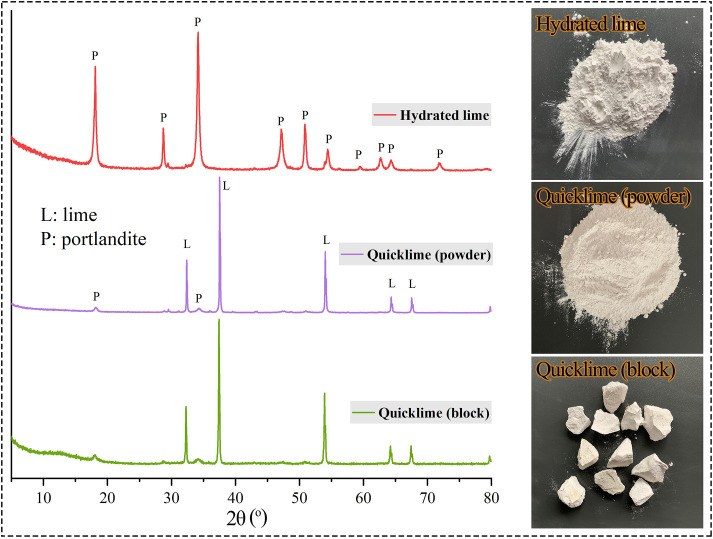
XRD pattern (left) and photograph (right) of the three types of lime used in the study.

The quicklime (both powdered and blocky forms) and hydrated lime were purchased from Sinopharm Chemical Reagent Co., Ltd. XRD analysis (Fig 3) showed that the hydrated lime was predominantly Ca(OH)_2_, while both quicklime types were mainly composed of CaO and Ca(OH)_2_. No signals of CaCO_3_ were identified in any of the three materials. The RIR semi-quantitative analysis (Table 1) indicated comparable CaO contents between the blocky (86.3%) and powdered (84.8%) quicklime, confirming their similar purity. The blocky quicklime was crushed to a 1–2 cm fraction for use. All experiments utilized deionized water.

### Sample preparation

In this study, the lime content was varied from 10% to 20% by mass.

The lime-soil mixtures were prepared by first dry-mixing the lime and soil in predetermined proportions. An appropriate amount of deionized water was then added and mixed thoroughly. Finally, the mixture was sealed and stored for 5 d. The specific mix proportions were provided in Table 2.

**Table 2:** Lime-soil mixture ratio (mass ratio).

Serial number	Lime			Soil
	Quicklime (block)	Quicklime (powder)	Hydrated lime	
1-1	10			90
1-2	15			85
1-3	20			80
2-1		10		90
2-2		15		85
2-3		20		80
3-1			10	90
3-2			15	85
3-3			20	80

According to different performance test requirements, two types of cylindrical specimens (diameter of 4 cm) were prepared using a light-weight tamping hammer [43]: 1) 90 g of lime-soil mixture compacted in two layers (25 hammer blows/layer); 2) 45 g of lime-soil mixture compacted in a single layer (25 hammer blows). Following demolding, the specimens were cured in the laboratory environment (T = 22 ± 3°C, RH = 75 ± 5%). To promote carbonation, the specimens were misted with water once per week.

### Property testing

#### Fresh lime-soil mixture.

1) The maximum dry density was determined through compaction tests [43]. For each mixture, an adequate amount of dry lime-soil was mixed with water at contents ranging from 14% to 30% by weight to ensure uniformity. After sealing and storing for 24 h, 135 g of the mixture was compacted in a mold (4 cm inner diameter) in three layers, with each layer receiving 25 blows from a light-weight tamping hammer. The height of the compacted specimen was measured with a caliper to calculate the dry density.
2) The temperature variation of the lime-soil mixture after adding water was monitored using a temperature recorder (HEEYII, HY-R3000). A 1 kg dry mixture was thoroughly mixed with an appropriate amount of water, transferred to an insulated foam box, and gently compacted. The probe of the temperature recorder was inserted into the center of the mixture, and temperature data were automatically logged at 5-second intervals for 10 h. The temperature recorder had a measurement range of −30–400°C.
3) The cohesion of the lime-soil mixture after adding water was assessed by a sieving method. The mixture was passed through a standard sieve, and the mass of aggregated particles retained on the sieve was measured. The content of aggregates in different size fractions was expressed as a mass percentage.

#### Cured lime-soil samples.

1) The specific surface area (SSA) and cation exchange capacity (CEC) of the cured lime-soil were determined by methylene blue titration [44,45]. Samples cured for 28 d were crushed, sieved through a 0.425 mm mesh, and 20 g of the dried powder was mixed with 200 ml deionized water to form a suspension. Methylene blue solution (5 g/L) was added in 2 ml increments under continuous stirring. After each addition, a droplet of the suspension was placed on filter paper; the titration endpoint was reached when a persistent blue halo formed around the soil aggregates. The total volume of methylene blue consumed was used to calculate SSA and CEC according to the following equations:

$$SSA=\frac{m_{MB}}{\text{3}\text{1}\text{9}.\text{8}\text{7}}A_{V}A_{MB}\frac{\text{1}}{m_{s}}$$ (1)

Where *m*_*MB*_ is the mass of adsorbed methylene blue, *A*_*V*_ is Avogadro’s constant (6.02 × 10^23^/mol), *A*_*MB*_ is the area covered by one methylene blue molecule (130 Å²), and *m*_*S*_ is the mass of the soil sample.


$$\begin{array}{c} CEC\left(\frac{cmol}{kg}\right)=\frac{\text{1}\text{0}\text{0}}{m_{s}}\times V_{cc}\times c \end{array}$$ (2)

Where $V_{cc}$ is the volume of methylene blue solution (ml) and cis its molar concentration (mol/L). All titrations were carried out in triplicate.

2) The water absorption coefficient was measured according to the standard [46]. Oven-dried specimens (55°C) were placed on a saturated permeable stone in a flat dish with the water level just below the top of the stone. Mass gain was recorded over time. The water absorption coefficient was determined from the slope of the mass gain versus the square root of time curve, divided by the test surface area.
3) The compressive strength was measured using a universal tensile and compression testing machine (model ZQ-970B, Hangzhou Qitai Technology Co., Ltd.). The specimens were tested after 28 and 60 d of curing. Three replicates were tested for each group, and the average value and standard deviation are reported.
4) The water resistance and freeze-thaw resistance of the specimens were qualitatively assessed based on their appearance. Following a 60-day curing period, the durability of the specimens was assessed. Water resistance was tested by immersing intact specimens in water for 28 d and observing their final condition. The freeze-thaw resistance test followed a modified procedure based on JGJ/T70-2009 [47]. Specimens were first saturated by capillary action on a permeable stone, and the surface water was wiped off before freezing. Each cycle consisted of freezing at −30°C for 12 h, followed by thawing in air at room temperature for 8 h. The thawing method was modified from the standard to prevent water-induced damage to the low-strength lime-soil during the thawing phase. The rating was defined as the number of cycles completed before observable failure occurred.

### Component analysis

Fourier Transform Infrared Spectroscopy (FT-IR) analysis was performed using a PerkinElmer Spectrum3 spectrometer equipped with a diamond attenuated total reflection (ATR) accessory to investigate the hydration process. Two types of lime-soil mixtures (one with blocky quicklime and one with powdered quicklime) were prepared by adding water, then sealed and stored. For the blocky quicklime-soil mixture, white particles (approximately 4−6 mm in diameter) were extracted at 0.5 h, 6 h, 2 d, and 5 d, dried at 200°C for 15 min, and analyzed separately from the edge and center. For the powdered quicklime-soil mixture, bulk samples were taken at 6 h and 24 h and similarly dried. All spectra were collected from 350 to 4000 cm^-1^ with 32 scans per spectrum at a resolution of 8 cm^-1^.

The microstructural characteristics of the lime-soil composites were investigated by scanning electron microscopy (SEM, ZEISS Gemini360). After 60 d of curing, specimens were fractured to expose fresh surfaces, which were then sputter-coated with gold and imaged.

The degree of carbonation of the lime-soil samples was assessed using a phenolphthalein reagent solution (0.5% w/w). The freshly split cross-section, parallel to the sample base, was tested at 1 mm depth intervals, and the color response was recorded.

No specific permits were required for this field study as it involved non-documented surface survey on public land with no access restrictions, and it did not involve any collection of protected cultural artifacts.

## Results and discussion

### Properties of fresh lime-soil mixture

#### Maximum dry density.

**Fig 4:**
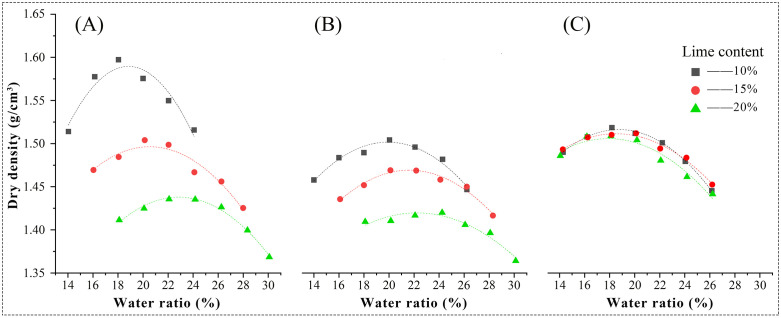
Dry density of different lime-soils ((A) Blocky quicklime; (B) Powdered quicklime; (C) Hydrated lime).

The maximum dry density test results were shown in Fig 4. Overall, for lime-soil prepared with both types of quicklime, the maximum dry density decreased with increasing lime content, while the optimum water content increased, consistent with previous literature [48,49]. This behavior was attributed to the higher proportion of fine, lightweight particles in the mixture [50]. The extent of this effect, however, varied with lime type: blocky quicklime had the strongest influence, followed by powdered quicklime. At a lime content of 10%, lime-soil prepared with blocky quicklime showed the highest maximum dry density (1.70 g/cm^3^). As the lime content increased, the maximum dry density decreased rapidly, dropping to 1.43 g/cm^3^ at 20% lime content (Fig 4A). Lime-soil prepared with powdered quicklime showed a similar but less pronounced trend, starting near 1.60 g/cm^3^ at 10% content (Fig 4B). In contrast, hydrated lime caused no significant change in maximum dry density or optimum water content across the tested range (Fig 4C). These differences were likely due to water consumption and evaporation during the exothermic hydration of quicklime.

#### Temperature change.

**Fig 5:**
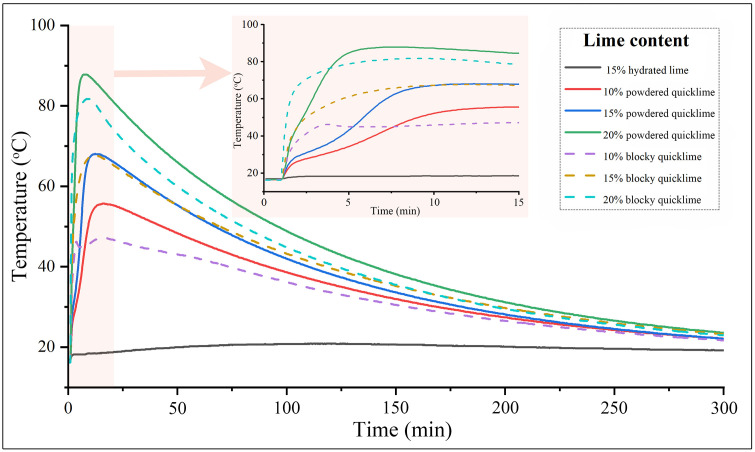
Temperature variation curves of different lime-soils during hydration.

The heat release profiles during the hydration of quicklime-soil mixtures were shown in Fig 5. As expected, higher quicklime content led to higher peak temperatures. However, the thermal behavior was distinctly governed by the lime’s physical form. Powdered quicklime, with its high specific surface area and uniform distribution, underwent rapid and simultaneous hydration, resulting in a high-intensity exothermic peak. In contrast, blocky quicklime exhibited a more complex profile. Its heterogeneous distribution caused intense local heating, leading to a faster initial temperature rise. However, the low specific surface area of the coarse particles and decelerated water diffusion to the unreacted core slowed the overall kinetics and limited the total heat release. This explained the lower peak temperatures and shorter durations of elevated temperatures observed for blocky quicklime at 10% and 20% contents. These thermal characteristics were critical for performance, as research demonstrated that both higher internal temperatures and extended periods of heat significantly enhance the properties of lime-soil [8,51].

#### Cohesion particle size.

**Fig 6:**
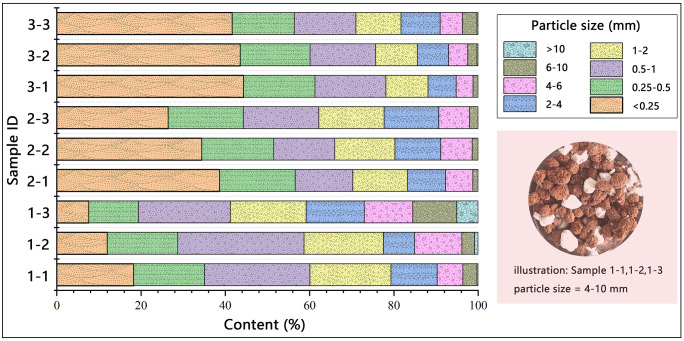
Distribution of lime-soil particles after mixing dry materials with water for 5 days.

The particle size distribution of lime-soil mixed with water after 5 d (Fig 6) revealed distinct agglomeration behavior. All mixtures showed increased particle cohesion with higher lime content, but the extent and nature of aggregation varied. Blocky quicklime caused the most significant agglomeration, with over 27% of particles exceeding 4 mm in size at 20% content. Approximately one-quarter of these large aggregates were undispersed lime lumps, indicating incomplete hydration and a heterogeneous microstructure. Powdered quicklime also enhanced the cohesion of lime-soil, though its effect was weaker than that of blocky quicklime; the formation of coarse particles (>4 mm) was not proportional to the lime content. Hydrated lime had the weakest effect, with about 40% of soil particles remaining unchanged at low content and only modest agglomeration occurring at higher contents. These differences in agglomeration were attributed to the physical state and reactivity of the different lime forms.

### Properties of cured lime-soil specimens

#### Compressive strength.

**Fig 7:**
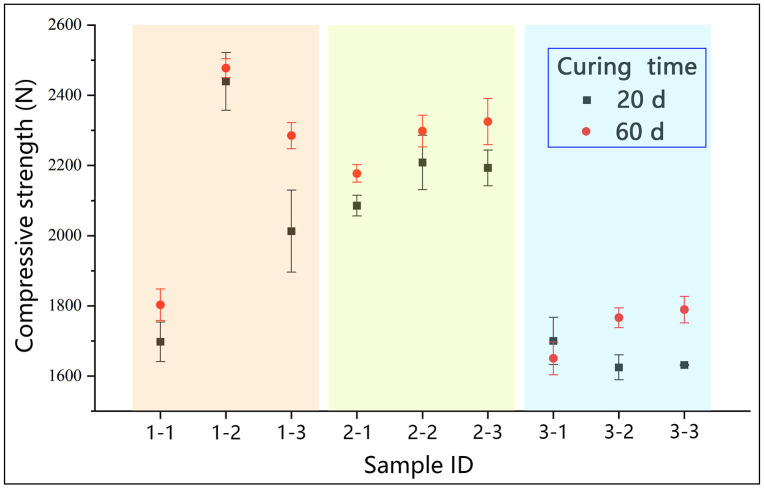
Compressive strength test results of different lime-soil specimens after 28-day and 60-day curing.

The compressive strength results of different lime-soil specimens were presented in Fig 7. Among the three types of lime, hydrated lime-soil exhibited the lowest strength, reaching only about 75% of the values achieved with quicklime-soil, and showed little improvement with increasing lime content. In contrast, powdered quicklime provided consistent, dosage-dependent strength gains, which was consistent with existing literature [18]. The most distinctive behavior was exhibited by blocky quicklime, which showed a pronounced optimum at 15% content, with strength declining at higher doses. This non-linear performance was likely related to its structural characteristics. At low content (10%), the incomplete dispersion and poor uniformity (as evidenced by the cohesion test in Fig 6) likely resulted in insufficient stabilization. However, at high content (20%), the decline in strength might be attributed to expansion stresses generated by the continued hydration of the oversized lime lumps during the curing period, as will be discussed in the subsequent microstructural analysis. Additionally, compressive strength was positively correlated with curing time for all mixtures, attributable to the continuous carbonation process of lime within the lime-soil mixtures [52].

#### Water absorption.

**Fig 8:**
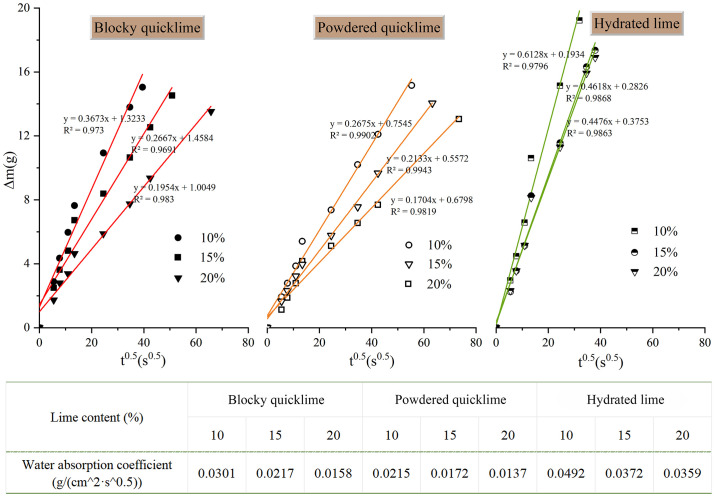
Water absorption test results of different lime-soil specimens.

The test results of water absorption for different lime-soil specimens were listed in Fig 8. Overall, the water absorption coefficients of all lime-soil specimens decreased with increasing lime content. When the lime content rose from 10% to 20%, the water absorption of specimens prepared with blocky quicklime, powdered quicklime, and hydrated lime decreased by 90%, 57%, and 37%, respectively. Moreover, under identical conditions, powdered quicklime resulted in the lowest water absorption, indicating superior waterproofing performance.

#### Water resistance.

**Fig 9:**
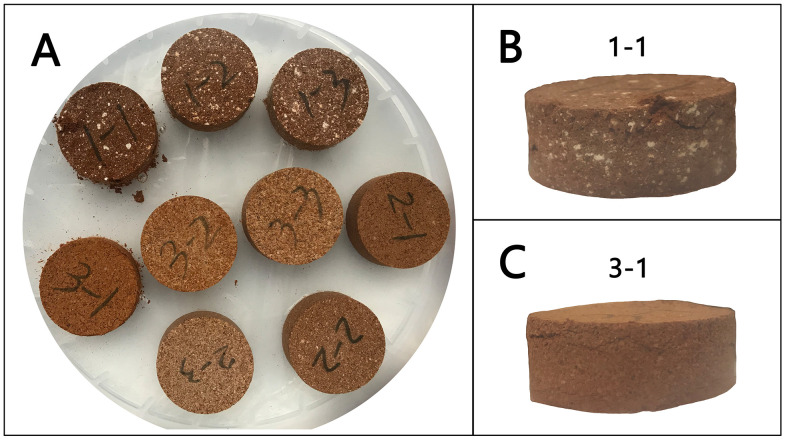
Photos of different lime-soil specimens after being soaked in water for 28 days.

The condition of various lime-soil specimens after 28 d of water immersion was illustrated in Fig 9. Visual assessment revealed that all specimens containing powdered quicklime, regardless of lime content, remained intact (Fig 9A). Conversely, specimens with 10% blocky quicklime (Fig 9B) or 10% hydrated lime (Fig 9C) exhibited significant damage (swelling, corner peeling, and cracking). At higher lime contents (≥15%), all specimens, including those with blocky and hydrated lime, showed no visible damage. This might be attributed to the weak stabilization ability of hydrated lime [18] and the incomplete dispersion of blocky quicklime (Fig 6), which formed localized weak zones. Therefore, the images supported the conclusions that powdered quicklime was the most effective type, and that blocky and hydrated lime required a higher content (≥15%) to achieve satisfactory performance.

#### Freeze-thaw resistance.

**Fig 10:**
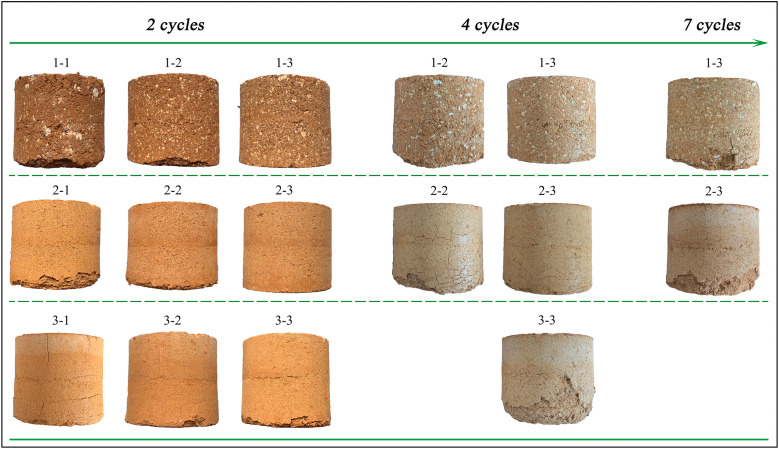
Photos of different lime-soil specimens after freeze-thaw cycles (*Note: Apparent color variations between specimens are due to differences in photographic lighting conditions and are not related to material chemical changes.*).

The freeze-thaw resistance test results were presented in Fig 10. The photographic sequence showed the maximum number of cycles each formulation could endure before failure. In general, lime-soil specimens prepared with quicklime (both blocky and powdered) exhibited superior freeze-thaw resistance. Although freeze-thaw resistance improved with higher lime content for all lime types, the failure mode and cycle count differed. At every content level (10%, 15%, 20%), hydrated lime-soil specimens failed earlier and with more severe damage (e.g., extensive cracking and swelling) than their quicklime-soil counterparts, which exhibited minor peeling even after enduring more cycles. This difference is critical for heritage conservation, as the ability to withstand freeze-thaw cycles is a key determinant of long-term durability for earthen structures in cold, seasonally frozen regions. Moreover, the performance between the two quicklime types (blocky and powdered) was comparable, with no significant difference observed.

### Structure and composition analyses

#### Specific surface area and cation exchange capacity.

**Fig 11:**
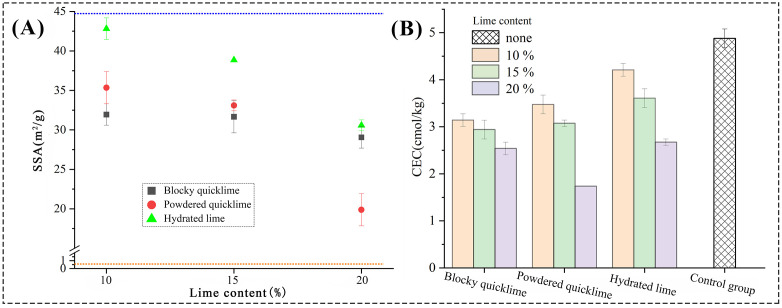
Test results of (A) specific surface area and (B) cation exchange capacity of different lime-soils.

The specific surface area (SSA) and cation exchange capacity (CEC) of the cured lime-soil were shown in Fig 11. Compared to pure soil (SSA: 44.6 m^2^/g), all lime types reduced the SSA and CEC, thereby inhibiting the soil’s natural sensitivity to water [53]. However, the effectiveness varied. Powdered quicklime caused the most pronounced reduction in SSA with increasing content, reaching 19.8 m^2^/g at 20% lime (Fig 11A). Blocky quicklime rapidly reduced the SSA to about 70% of pure soil at 10% content, with diminishing returns thereafter. Hydrated lime was the least effective.

The CEC results (Fig 11B) provided a mechanistic explanation for the performance trends observed in the water resistance tests (Fig 9). A lower CEC signified stronger soil flocculation and reduced ability to absorb water, leading to better stability [54]. This was crucial for relic protection because a low CEC directly translated to reduced water uptake and swelling pressure, which were primary causes of erosion and deterioration in ancient earthen structures [55,56]. The more effective CEC reduction by quicklime, especially the powdered form, explained their better performance in creating water-resistant lime-soil materials. Furthermore, the low CEC and SSA of the powdered quicklime mixtures also correlated with their excellent freeze-thaw resistance (Fig 10). By minimizing water infiltration and creating a stable, aggregated soil structure, these materials were better to resist the destructive cycles of freezing and thawing. This property was particularly crucial for the long-term preservation of outdoor architectural heritage located in cold and seasonally frozen regions.

#### Microstructure.

**Fig 12:**
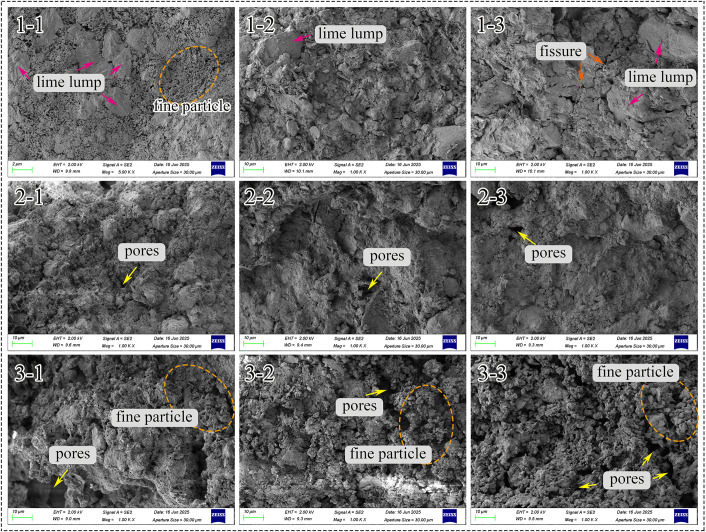
The SEM pictures of different lime-soil specimens after curing for 60 days.

SEM images after 60-day curing (Fig 12) revealed distinct microstructural features that correlate with the observed macroscopic properties. Lime-soil specimens prepared with blocky quicklime showed angular, partially hydrated lime lumps. The specimen with 10% lime content (*1–1*) showed fine particles and numerous pores, explaining its low strength and poor durability, while coarser, bonded structures formed at higher contents (*1–2* and *1–3*). Microcracks were observed at 20% content (*1–3*), likely contributing to the measured strength reduction at this dosage. In contrast, specimens made with powdered quicklime (*2–1*, *2–2*, and *2–3*) exhibited a consistent, dense structure with minimal porosity, directly corresponding to their superior waterproofing and mechanical performance. Lime-soil specimens containing hydrated lime showed loose, porous structures with abundant fine particles, aligning with their high SSA and inferior properties. These structural observations provided direct evidence for the performance variations measured in mechanical and durability tests.

#### Hydration process.

**Fig 13:**
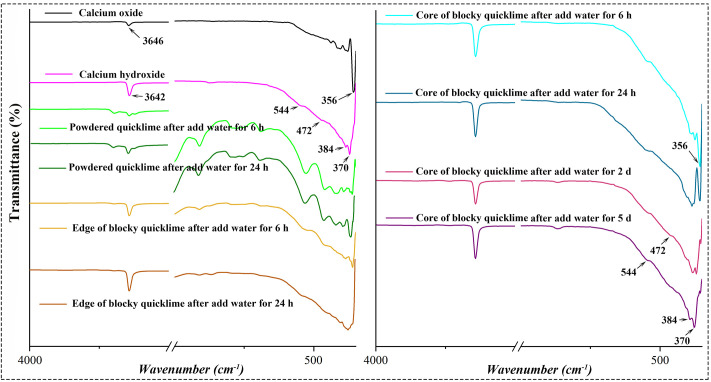
FTIR spectra of pure calcium oxide, pure calcium hydroxide, and lime-soil mixtures after adding water for certain periods.

The hydration process of quicklime in the lime-soil mixture was monitored using infrared spectroscopy, as illustrated in Fig 13. The infrared spectra revealed that calcium oxide exhibited characteristic vibrations within the range of 300−600 cm^-1^, with multiple fine structures. Notably, a sharp Ca-O bending vibration band was observed at 356 cm^-1^ [57]. Calcium hydroxide also displayed several characteristic Ca-O vibration bands within the same range, including two broad bands at 544 and 472 cm^-1^, and two sharp bands at 384 and 370 cm^-1^. Additionally, both calcium oxide and calcium hydroxide exhibited an absorption band near 3645 cm^-1^, corresponding to the stretching vibration of hydroxyl groups on the surface of calcium oxide and within calcium hydroxide [58].

In the lime-soil prepared with powdered quicklime, the characteristic band of calcium oxide (356 cm^-1^) was detectable in the sample at 6 h. This band disappeared after 24 h, indicating complete hydration of the powdered quicklime. In contrast, for lime-soil prepared with blocky quicklime, water caused the blocky quicklime to decompose into particles of varying sizes. After 24 h, all surface calcium oxide has converted to calcium hydroxide. However, within larger particles (diameter > 4 mm), the characteristic peak of calcium oxide (356 cm^-1^) gradually weakens over time, while the characteristic peaks of calcium hydroxide (384 and 370 cm^-1^) gradually strengthen. Notably, even after 5 d, the characteristic absorption band of calcium oxide (356 cm^-1^) within these larger particles didn’t fully disappear.

The subsequent hydration of these unhydrated lime lumps during extended curing might present a dual effect on the lime-soil’s long-term performance. On one hand, the associated volumetric expansion [59] could potentially densify the matrix and contribute to self-healing capacity by filling pre-existing microcracks, a phenomenon documented in lime-based mortars [22]. On the other hand, as observed in lime-soil specimen made with 20% blocky quicklime (Fig 12*(1–3)*), excessive expansion stress generated by a high proportion of these lumps is detrimental, leading to microcrack formation and ultimately explaining the measured decline in compressive strength [60]. This underscores the necessity to carefully control the dosage of blocky quicklime to harness its potential benefits while mitigating the risks of internal damage.

#### Carbonation process.

**Fig 14:**
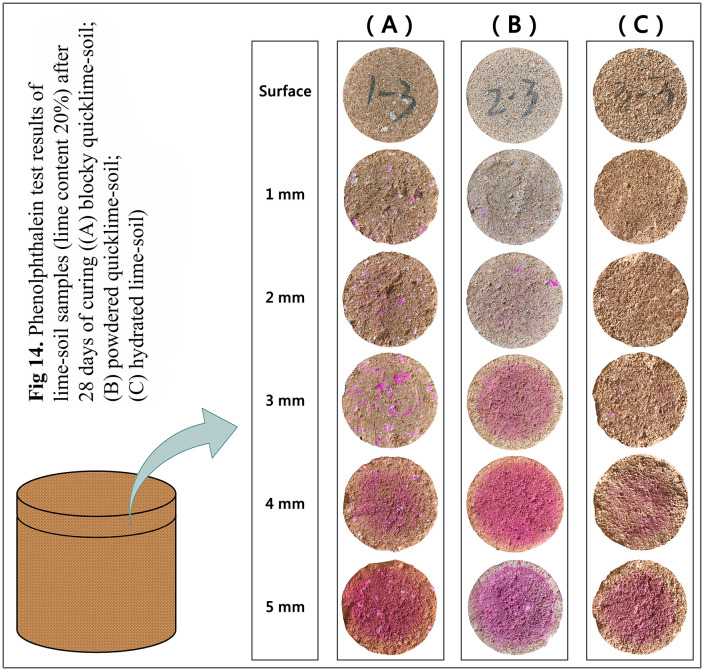
Phenolphthalein test results of lime-soil specimens (lime content 20%) after 28 days of curing ((A) blocky quicklime-soil; (B) powdered quicklime-soil; (C) hydrated lime-soil).

The carbonation depths of lime-soil specimens with 20% lime content after 28 d of curing were shown in Fig 14. Phenolphthalein tests revealed a distinct carbonation gradient: hydrated lime > blocky quicklime > powdered quicklime. In lime-soil specimen made with hydrated lime (Fig 14C), a very light pink color was visible at a 4 mm depth. For specimen made with blocky quicklime (Fig 14A), pink staining was mainly observed on larger lime lumps at a 3 mm depth. At 4 mm, the central area showed pink coloration, but with weaker intensity and smaller coverage area compared to specimen made with powdered quicklime (Fig 14B). This pattern appeared to be influenced by the microstructure observed via SEM (Fig 12). The loose, porous nature of the specimen made with hydrated lime likely facilitated deeper CO_2_ penetration, whereas the dense, cohesive matrix of the quicklime-soil mixture impeded it. Moreover, the specimen with the deepest carbonation (hydrated lime) exhibited the lowest compressive strength (Fig 7). This inverse relationship suggested that in this study, factors such as ion exchange efficiency and the formation of a cohesive microstructure—governed by the lime form—had a more significant impact on early strength development of lime-soil mixture than the carbonation process itself.

## Conclusion

This work investigated the performance of lime-soil materials prepared with blocky quicklime, powdered quicklime, and hydrated lime. Through lab simulations of lime-soil performance tests and material structure and composition analyses, the following conclusions were drawn:

(1) The chemical form of lime was a critical performance determinant, significantly influencing the properties of both the fresh and cured mixtures. In the fresh state, the exothermic hydration of quicklime enhanced early particle cohesion and aggregation, as evidenced by particle size distribution analysis. In the cured state, hydrated lime exhibited weaker ionic exchange capacity and resulted in a loose, porous microstructure, leading to inferior strength and durability. In contrast, both types of quicklime significantly reduced the soil’s specific surface area (SSA) and cation exchange capacity (CEC), promoting the formation of a denser matrix that improved engineering properties.
(2) Powdered quicklime proved to be the most reliable and effective choice. It provided consistent, dosage-dependent improvements in compressive strength, water resistance, and freeze-thaw resistance. Its rapid and complete hydration resulted in a uniform and dense microstructure (SEM), which underpinned its superior performance. For high-performance requirements in heritage repair, its application at 15–20% content was recommended.
(3) Blocky quicklime required precise dosage control. It exhibited a distinct optimum at 15% content, facilitating the formation of a dense matrix. However, exceeding this optimum led to a performance decline. Structure and composition analyses revealed that this was due to expansion stresses from excess: partially hydrated lime lumps (as identified by FT-IR), which generated micro-cracks (as observed by SEM) at higher dosages. Therefore, its application must be carefully calibrated.
(4) This work constituted preliminary laboratory-scale research and didn’t fully simulate the complex environmental conditions of actual heritage sites. Therefore, future work should focus on field validation and monitoring of the reaction processes. In addition, while this study provided short-term performance data (60 d) for lime-soil mixtures, the long-term performance of systems prepared with different lime types (such as the self-healing potential of blocky quicklime-soil systems) required further investigation. Such efforts would help scientifically elucidate the sustainability and rationality of traditional techniques, thereby providing more comprehensive support for the sustainable conservation of cultural heritage.
